# Biphasic Cholinergic Modulation of Reverberatory Activity in Neuronal Networks

**DOI:** 10.1007/s12264-022-01012-7

**Published:** 2023-01-21

**Authors:** Xiao-Wei Li, Yi Ren, Dong-Qing Shi, Lei Qi, Fang Xu, Yanyang Xiao, Pak-Ming Lau, Guo-Qiang Bi

**Affiliations:** 1 CAS Key Laboratory of Brain Function and Disease, School of Life Sciences, Division of Life Sciences and Medicine, University of Science and Technology of China, Hefei, Anhui 230027 China; 2 CAS Key Laboratory of Brain Connectome and Manipulation, Interdisciplinary Center for Brain Information, the Brain Cognition and Brain Disease Institute (BCBDI), Shenzhen Institute of Advanced Technology, Chinese Academy of Sciences; Shenzhen-Hong Kong Institute of Brain Science-Shenzhen Fundamental Research Institutions, Shenzhen, 518055 China

**Keywords:** Acetylcholine, Neuronal network, Reverberation, Synaptic current, Excitability

## Abstract

Acetylcholine (ACh) is an important neuromodulator in various cognitive functions. However, it is unclear how ACh influences neural circuit dynamics by altering cellular properties. Here, we investigated how ACh influences reverberatory activity in cultured neuronal networks. We found that ACh suppressed the occurrence of evoked reverberation at low to moderate doses, but to a much lesser extent at high doses. Moreover, high doses of ACh caused a longer duration of evoked reverberation, and a higher occurrence of spontaneous activity. With whole-cell recording from single neurons, we found that ACh inhibited excitatory postsynaptic currents (EPSCs) while elevating neuronal firing in a dose-dependent manner. Furthermore, all ACh-induced cellular and network changes were blocked by muscarinic, but not nicotinic receptor antagonists. With computational modeling, we found that simulated changes in EPSCs and the excitability of single cells mimicking the effects of ACh indeed modulated the evoked network reverberation similar to experimental observations. Thus, ACh modulates network dynamics in a biphasic fashion, probably by inhibiting excitatory synaptic transmission and facilitating neuronal excitability through muscarinic signaling pathways.

**Supplementary Information:**

The online version contains supplementary material available at 10.1007/s12264-022-01012-7.

## Introduction

The brain’s cognitive functions, such as learning and memory, are carried out by coordinated activity in complex neuronal circuits that are profoundly influenced by various neuromodulators [1, 2]. One of the most important and widespread neuromodulators is acetylcholine (ACh). The cholinergic system originating from subcortical regions innervates many brain areas, including the cortex and the hippocampus [3], and plays important roles in arousal, attention, and learning [4]. It has long been known that the muscarinic ACh receptor antagonist scopolamine impairs learning in both humans and animals [5, 6]. In particular, intravenous administration of scopolamine impairs spatial working memory independent of sensorimotor and procedural learning deficits [7]. Disruption of cholinergic projections from the basal forebrain nucleus basalis magnocellularis to the neocortex or the hippocampus also impairs learning and memory in rats and monkeys [8–10]. Furthermore, severe loss of cholinergic neurons in the nucleus basalis in Alzheimer’s disease has been proposed to contribute to the memory deficit in patients [11, 12]. It is thus of great interest to understand at the molecular level how the action of ACh on its receptors eventually leads to changes in system-level functions.

In addition to the pharmacological- and behavioral-level evidence for the important role of ACh in learning and memory, electrophysiological studies have revealed that ACh release elicits or facilitates neural synchrony in the cortex and theta oscillations in the hippocampus [13–15]. Optogenetic activation of septal cholinergic neurons disrupts hippocampal ripple oscillations but enhances theta oscillations *via* muscarinic receptors [16, 17]. Interestingly, neural oscillations, especially theta oscillation, have been shown to be intimately associated with working memory, as well as the encoding and retrieval of episodic memory in humans [18, 19]; such oscillations in neuronal ensembles may provide temporal reference frames for brain information encoding and facilitate synaptic plasticity [20, 21]. At the cellular level, it is well known that ACh can alter synaptic transmission *via* both pre- and postsynaptic muscarinic receptors [22, 23], and can regulate K^+^ channels, thereby changing neuronal excitability [24, 25]. However, understanding exactly how such molecular and cellular actions of ACh translate into modulation of the network activity of neuronal ensembles *in vivo* has been challenging, in part due to the vast complexity of native circuits.

Previously, we have demonstrated that small networks of cultured hippocampal neurons exhibit persistent activity in response to brief stimuli applied to single cells [26], reminiscent of reverberatory activity in the hypothetical cell assembly proposed by Donald Hebb as a network basis of “online” working memory [27]. Such network reverberation is driven by recurrent excitation, sustained by asynchronous synaptic transmission, and terminated by a slow component of short-term synaptic depression [26, 28–30]. This *in vitro* model system, by virtue of its simplicity and accessibility, provides a unique opportunity for examining the network effects of neuromodulation, as well as the underlying cellular mechanisms. In the current work, we investigated the influence of different levels of ACh on evoked reverberatory activity as well as spontaneous activity in cultured neuronal networks, and found that ACh modulates network activity in a biphasic fashion. We further explored the cellular mechanisms underlying such biphasic modulation with whole-cell patch-clamp recording of synaptic currents and excitability, pharmacological manipulations of nicotinic and muscarinic ACh receptors, and computational modeling.

## Materials and Methods

### Cell Culture

All procedures were performed following the guidelines and protocols approved by the Animal Experiments Committee of the University of Science and Technology of China.

Primary rat hippocampal cultures were prepared according to a previously described protocol [31] with modification. Hippocampi were dissociated from embryonic day 18 rats, treated with 0.25% trypsin (Sigma-Aldrich, St. Louis, USA) at 37°C for 15 min, gently washed with Hank’s balanced salt solution buffer (Thermo Fisher, Waltham, USA), and then triturated with a fire-polished glass pipette in culture medium. These suspended cells were plated on glass coverslips in 35-mm Petri dishes with ~40,000 to 80,000 cells/mL. Coverslips were pre-coated with poly-L-lysine (Sigma-Aldrich), and formed patterns of spots (1–1.5 mm in diameter) by using custom-made stamps. Apart from the island coating, we also made some whole coatings by immersing the coverslips in poly-L-lysine. The culture medium was Dulbecco’s modified Eagle’s medium (BioWhittaker, Basel, Switzerland) supplemented with 5% heat-inactivated fetal bovine serum (HyClone, Logan, USA) and 5% heat-inactivated bovine calf serum (PAA Laboratories, Pasching, Austria), 10% Ham’s F-12 with glutamine (BioWhittaker), 50 units/mL penicillin/streptomycin (Gibco, Grand Island, USA), and 2% B-27 supplement (Thermo Fisher). Cultures were then incubated at 37°C under 7% CO_2_. Approximately 24 h after plating, one-third of the culture medium was replaced by the same medium supplemented with KCl (20 mmol/L) to promote the growth of neuronal protrusions. At 7–9 days *in vitro* (DIV), cytosine arabinoside (Sigma-Aldrich) was added to the culture medium (final concentration, 1–5 μmol/L) to prevent the overgrowth of glial cells. Cultures were used at 9–18 DIV for electrophysiological recording. Glial cells were restricted in island-coated poly-L-lysine spots, and neurons tended to grow on the glial cell islands. The whole-coated coverslips led to large neuronal networks that had more spontaneous activity.

### Electrophysiology

Perforated whole-cell patch clamp was carried out at room temperature with patch-clamp amplifiers (MultiClamp 700B, Axon Instruments, San Jose, USA). The pipette solution contained (in mmol/L): 136.5 potassium gluconate, 17.5 KCl, 9 NaCl, 1 MgCl_2_, 10 HEPES, 0.2 EGTA, and 200 μg/mL amphotericin B (pH 7.3). The external bath solution contained (in mmol/L): 150 NaCl, 3 KCl, 3 CaCl_2_, 2 MgCl_2_, 10 HEPES, and 5 glucose (pH 7.3). All of these reagents were from Sigma-Aldrich. The drugs used were: acetylcholine hydrochloride (Sigma-Aldrich), mecamylamine hydrochloride (Sigma-Aldrich), scopolamine hydrobromide (Tocris, Bristol, UK), atropine sulfate monohydrate (Selleck, Houston, USA), carbamoylcholine chloride (Tocris), 6-cyano-7-nitroquinoxaline-2,3-dione (CNQX) (Tocris), bicuculline methiodide (BMI) (Tocris), and D(–)-2-amino-5-phosphonopentanoic acid (D-AP5) (Tocris). Acetylcholine hydrochloride powder was weighed immediately after opening, dissolved in water as 10 mmol/L stock solution, and then diluted in external bath solution to the final concentrations (0.01–100 μmol/L) in experiments. Stock solutions of other drugs were also prepared in water or DMSO, and diluted (1:1,000) in external bath solution when used. Throughout the patch clamp recording, the culture was perfused with bath solution at a constant rate of 1 mL/min. Signals were filtered at 2 kHz, and acquired at a sampling rate of 10 kHz using a 16-bit digitizing board (PCI-6229, National Instruments, Austin, USA) interfaced with custom Igor Pro (WaveMetrics, Portland, USA) programs. The pipette resistance was 2–3 MΩ. Data were accepted for analysis if the series resistance did not change >20% throughout the experiment.

For reverberation recording, voltage clamp was usually used, and all test stimuli (1.5 ms, 150 mV) were given to a single neuron at a fixed interval (30 s) to allow for network recovery. Networks showing systematic run-up or run-down in reverberation during the control period were excluded from further analysis. For monosynaptic current recording, excitatory postsynaptic currents (EPSCs) and inhibitory postsynaptic currents (IPSCs) were distinguished by their waveforms, in which the IPSCs had distinctly longer decay times (>20 ms) and more negative reversal potentials (approximately −50 mV) than EPSCs, and the specific blocker CNQX or BMI was applied for further confirmation. Trials showing systematic changes during the control period (>10% in 10 min) were excluded. For neuronal excitability measurements, a depolarizing current (500 ms, 100–400 pA) was injected to elicit ~4 spikes under current-clamp mode. CNQX (10 μmol/L), D-AP5 (25 μmol/L), and BMI (10 μmol/L) were applied to block synaptic transmission.

### Data Analysis

In each experiment, at least 20 consecutive traces were acquired under a given condition (e.g., before, ACh, and wash) to characterize reverberation (e.g., duration and occurrence probability). After the stimulation of one neuron, one or multiple polysynaptic currents (PSCs) above the threshold (100 pA) were recorded. A series of PSCs (at least two) with an inter-PSC-interval <500 ms formed persistent activity. The duration of persistent activity was defined as the time from the rising phase of the first PSC to the falling phase of the last PSC. Only trials with a duration >500 ms (it persisted and included at least two PSCs) and an initial latency <50 ms (it was induced by the pulse) were considered as evoked reverberation. Under a given condition, the probability of occurrence of reverberation was the count of evoked reverberation divided by the number of test stimuli, and the duration of reverberation was the mean value of duration for all evoked reverberation. The first PSC charge was the area under the first PSC event (suprathreshold PSCs with spikes were excluded from analysis). PSCs that occurred without stimulation were considered to be spontaneous, and multiple spontaneous PSCs that occurred continuously (i.e., inter-PSC-interval <500 ms) were identified as one episode of spontaneous reverberation. The average duration was the mean value of the durations of all episodes of spontaneous reverberation within a given segment(s) of recording. The rate or frequency of spontaneous reverberation was the number of spontaneous episodes per minute within a time period.

Comparisons were made using paired or unpaired two-tailed Student’s *t*-test. Significance is labeled as *P <0.05, **P <0.01, and ***P <0.001, and ^#^ is used for between-group comparisons. Values are reported as the mean ± SEM.

### Network Simulation

The simulation of reverberatory activity is based on previous work [28, 30]. We outline the neuronal network model here for convenience. Eq. (1) describes the membrane potential dynamics of a neuron; Eq. (2) describes the synaptic interaction; and Eq. (3) describes the synaptic vesicle cycle dynamics within a synapse.

1 $$ \left\{ \begin{gathered} C \frac{{{\text{d}}V_{i} }}{{{\text{d}}t}} = { } - g_{L} \left( {V_{i} - V_{L} } \right) - I_{{{\text{ion}},i}} \left( {V_{i} ,t} \right) + I_{{{\text{syn}},i}} + I_{{{\text{app}},i}} \left( t \right) + I_{{{\text{sti}},i}} \left( t \right) \hfill \\ I_{{{\text{ion}},i}} \left( {V_{i} ,t} \right){ } = { }g_{{{\text{Ca}}}} m_{\infty } \left( {V_{i} } \right)\left( {V_{i} - V_{{{\text{Ca}}}} } \right) + g_{K} W\left( {V_{i} ,t} \right)\left( {V_{i} - V_{K} } \right) \hfill \\ \frac{{{\text{d}}W\left( {V_{i} ,t} \right)}}{\text{d}t}{ } = { }\theta \frac{{W_{\infty } \left( {V_{i} } \right) - W\left( {V_{i} ,t} \right)}}{{\tau_{W} \left( {V_{i} } \right)}} \hfill \\ m_{\infty } \left( {V_{i} } \right){ } = { }\frac{1}{2}\left[ {1 + \tanh \left( {\frac{{V_{i} - V_{1} }}{{V_{2} }}} \right)} \right] \hfill \\ W_{\infty } \left( {V_{i} } \right){ } = { }\frac{1}{2}\left[ {1 + \tanh \left( {\frac{{V_{i} - V_{3} }}{{2V_{4} }}} \right)} \right] \hfill \\ \tau_{W} \left( {V_{i} } \right){ } = { }\left[ {\cosh \left( {\frac{{V_{i} - V_{3} }}{{2V_{4} }}} \right)} \right]^{ - 1} \hfill \\ \end{gathered} \right\} $$

2 $$ \begin{gathered} I_{{{\text{syn}},i}} { } = { } - g_{{{\text{syn}},i}} \left( {V_{i} - V_{E} } \right) \hfill \\ g_{{{\text{syn}},i}} { } = { }\mathop \sum \nolimits_{j} W_{i,j} Y_{i,j} \hfill \\ \end{gathered} $$

3 $$ \left\{ \begin{gathered} \frac{{{\text{d}}X}}{{{\text{d}}t}}{ } =  {\frac{S}{{\tau_{s} }} + \frac{Z}{{\tau_{r} }} - \lambda_{{{\text{EPSC}}}} uX\delta \left( {t - t_{{{\text{spike}}}} } \right)}  \hfill \\ \frac{{{\text{d}}Y}}{{{\text{d}}t}}{ } = { - \frac{Y}{{\tau_{d} }} + \lambda_{{{\text{EPSC}}}} uX\delta \left( {t - t_{{{\text{spike}}}} } \right)}  \hfill \\ \frac{{{\text{d}}Z}}{{{\text{d}}t}}{ } = {\frac{Y}{{\tau_{d} }} - \frac{Z}{{\tau_{r} }} - \frac{Z}{{\tau_{l} }}}  \hfill \\ \frac{{{\text{d}}S}}{{{\text{d}}t}}{ } =  {\frac{Z}{{\tau_{l} }} - \frac{S}{{\tau_{s} }}} \hfill \\ \end{gathered} \right. $$

In Eq. (3) *X*, *Y*, *Z*, and *S* are the fractions of synaptic resource states corresponding to the recovered, active, inactive, and super-inactive presynaptic vesicle pools. With constraint *X* + *Y* + *Z* + *S* = 1, *t*_spike_ in Eq. (3) represents the timing of presynaptic spikes. *I*_sti_ in Eq. (1) represents the stimuli. See Table S1 for the values and description of parameters in Eqs (1), (2), and (3).

*λ*_EPSC_ represents the increment of vesicle release when receiving a spike, and *g*_L_ represents the conductance of membrane leakage; they are the main factors influencing synaptic transmission strength and membrane excitability, respectively. When *λ*_EPSC_ declines, the evoked synaptic release decreases and reduces the EPSC amplitude. As *g*_L_ decreases, the excitability of each neuron increases. The manipulation of ACh concentration in the experiment induced changes in excitability and EPSC amplitude. Therefore, in the simulation, we tuned the neuronal excitability through *g*_L_, and the EPSC amplitude through *λ*_EPSC_. We then chose a set of parameters to mimic the experimental effect of ACh on these cellular properties and to evaluate the effects on simulated network reverberation.

In the model, parameters other than *λ*_EPSC_ and *g*_L_ may also affect the EPSC and excitability. However, *g*_L_ is the single strongest factor that affects excitability in the sense of having the largest partial derivatives of neuronal spiking rate with respect to all possible parameters. The partial derivative with respect to *g*_L_ is at least 4 times larger than all other parameters (Table S2), and if we tune other parameters for excitability, the values can easily go to the non-physiological regime while the excitability changes only a little. Changes in EPSC amplitude might also be accomplished by changing the network connectivity strength, i.e., *W*_*i,j*_ in Eq. (2), but the results generated by tuning *W*_*i,j*_ do not agree with the experimental observations.

## Results

### Biphasic Modulation of Evoked Network Reverberation by ACh

**Fig. 1:**
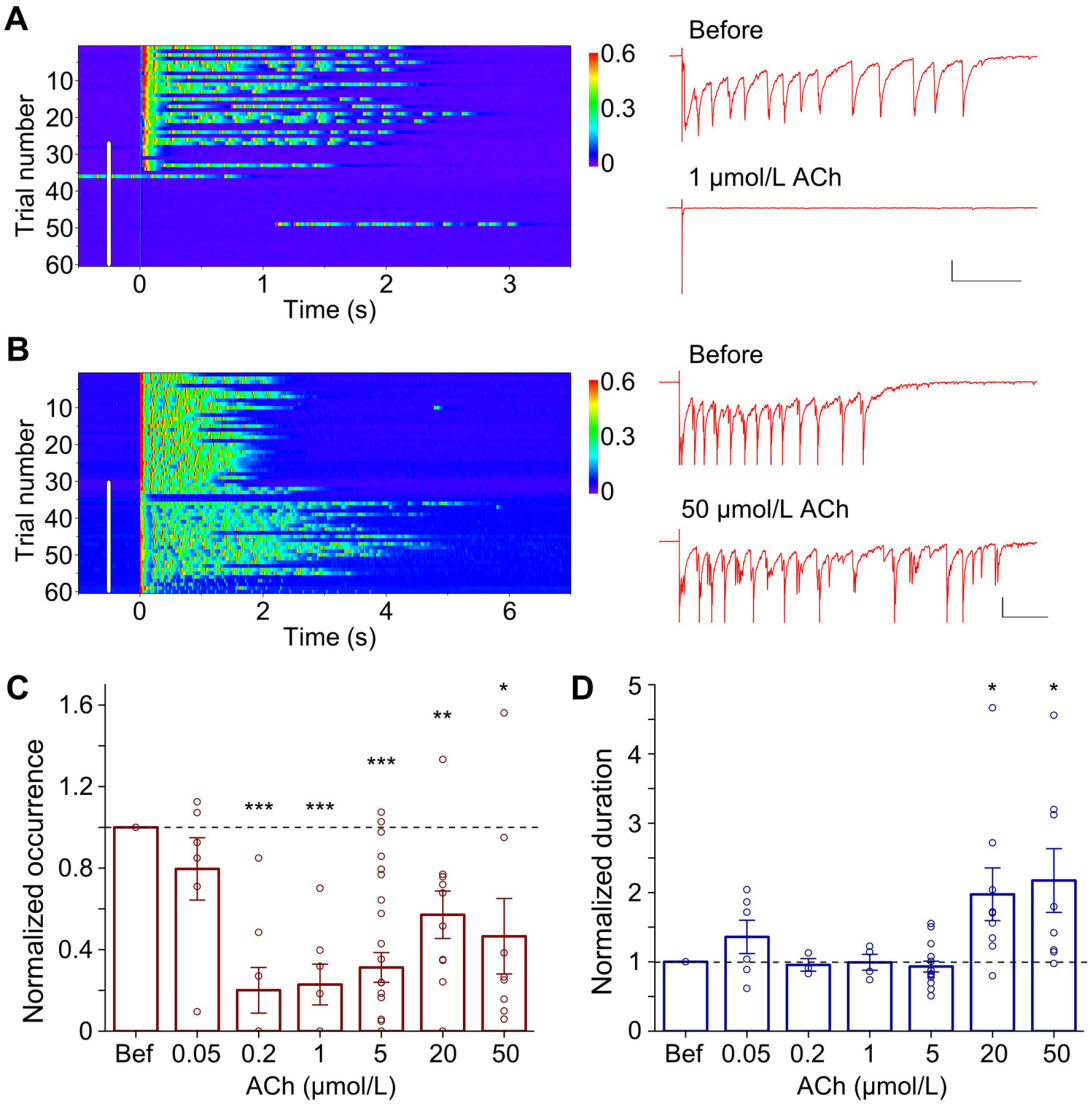
Differential dose-dependent effects of ACh on the occurrence and duration of evoked reverberation. **A** Suppression of evoked reverberation in the presence of ACh (1 μmol/L). Left: consecutive trials of current traces of one neuron, with the stimuli at time 0. Pseudo-color represents the current amplitude (in nA). The white vertical bar indicates the period when the ACh-containing solution was being perfused (the same for **B**). Right: example traces before and after adding ACh (1 μmol/L) an expanded view (scale bars, 0.2 nA and 0.5 s). **B** Enhancement in the duration of evoked reverberation with a high concentration of ACh (50 μmol/L). Scale bars, 0.2 nA and 0.5 s. **C** Effects of ACh (0.05–50 μmol/L) on the probability of reverberation occurrence. Each column is the mean value from a set of experiments for each ACh concentration normalized to the value before ACh application (Bef) (0.05 µmol/L: 0.80 ± 0.15, *n* = 6, *P* = 0.22; 0.2 µmol/L: 0.20 ± 0.11, *n* = 8, ***P <0.001; 1 µmol/L: 0.23 ± 0.10, *n* = 7, ***P <0.001; 5 µmol/L: 0.31 ± 0.07, *n* = 27, ***P <0.001; 20 µmol/L: 0.57 ± 0.11, *n* = 10, **P <0.01; 50 μmol/L: 0.47 ± 0.19, *n* = 8, *P <0.05. Paired *t*-test, ACh *vs* Bef). **D** Effects of ACh (0.05–50 μmol/L) on normalized reverberation duration (0.05 µmol/L: 1.36 ± 0.24, *n* = 6, *P* = 0.28; 0.2 µmol/L: 0.95 ± 0.09, *n* = 3, *P* = 0.68; 1 µmol/L: 0.99 ± 0.11, *n* = 4, *P* = 0.68; 5 µmol/L: 0.93 ± 0.08, *n* = 15, *P* = 0.12; 20 µmol/L: 1.97 ± 0.38, *n* = 9, *P <0.05; 50 μmol/L: 2.17 ± 0.46, *n* = 8, *P <0.05. Paired *t*-test, ACh *vs* Bef).

We made whole-cell patch-clamp recordings from one or two neurons in a cultured neuronal network (20–100 neurons). Single-pulse stimuli (1.5 ms, 150 mV) applied to a glutamatergic neuron at a low frequency (0.03 Hz) often elicited persistent network reverberation in the recorded cells [26]. In each of these networks, reverberation was induced in an all-or-none fashion, with relatively consistent occurrence probability and duration. With a low concentration of ACh (1 μmol/L) acutely added into the perfusion solution, the occurrence probability of reverberation was significantly decreased (Fig. 1A). The ACh receptor agonist carbachol had similar inhibitory effects on reverberation, which was reversed when the drug was washed out (Fig. S1). Intriguingly, compared to a low concentration, a high concentration of ACh (50 μmol/L) had less inhibition of the occurrence of reverberation (Fig. 1B). Furthermore, the duration of reverberation was significantly increased in the presence of 50 μmol/L ACh (Fig. 1B). Varying doses revealed that ACh influences reverberation in a biphasic fashion (Fig. 1C, D). Low-to-moderate doses of ACh (0.2–5 μmol/L) effectively inhibited the occurrence of evoked reverberation, but high doses (20–50 μmol/L) were less effective (Fig. 1C). Furthermore, while the duration of the reverberation was not affected by low-to-moderate doses, it was significantly increased by high doses of ACh (20–50 μmol/L) (Fig. 1D).

### High Doses of ACh Enhance Spontaneous Reverberation in Neuronal Networks

**Fig. 2:**
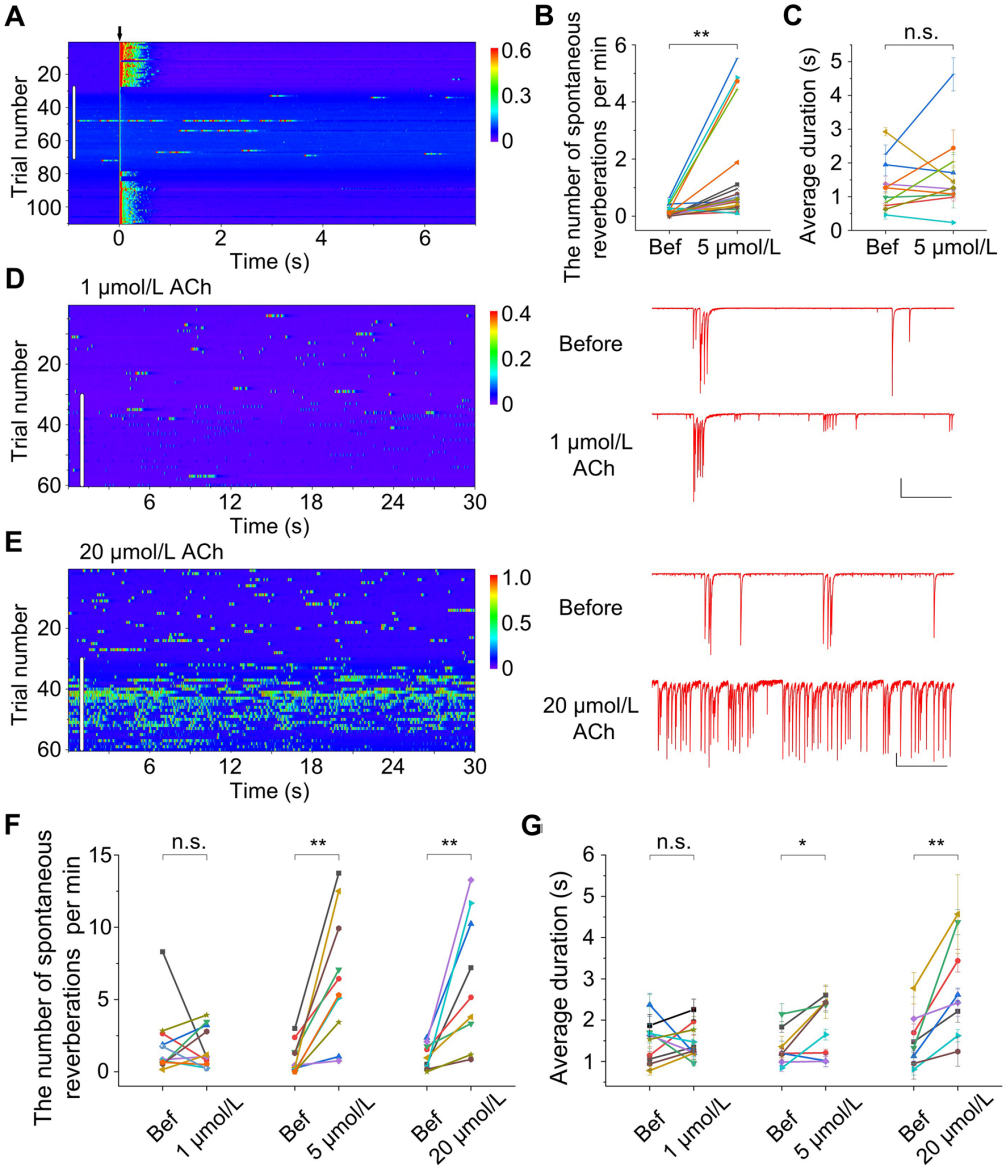
High-dose ACh increases spontaneous reverberation in neuronal networks. **A** Spontaneous reverberation is enhanced while evoked reverberation is reduced in an example network in the presence of 5 μmol/L ACh (white vertical bar). Single-pulse stimuli are delivered at time 0 every 30 s. Pseudo-color represents the current amplitude (in nA). The white vertical bar indicates the period when the ACh-containing solution was being perfused (the same for **D** and **E**). **B** The number of spontaneous reverberations per min is significantly enhanced with 5 μmol/L ACh. Each data point is the number of spontaneous reverberation per min before and after ACh application in one experiment, line segments of different colors represent different experiments (*P* <0.01, *n* = 22, paired *t*-test). Bef, before. **C** Average duration of spontaneous reverberation does not change significantly with 5 μmol/L ACh (*P* = 0.33, *n* = 11, paired *t*-test). **D**, **E** ACh effects on spontaneous reverberation in two example networks. Spontaneous reverberation does not change much with 1 μmol/L ACh (**D**) but is significantly enhanced with 20 μmol/L ACh (**E**). Scale bars, 0.2 nA, 5 s. **F** Summary of ACh effects on the number of spontaneous reverberations per min (1 μmol/L: *P* = 0.79, *n* = 11; 5 μmol/L: *P* <0.01, *n* = 10; 20 μmol/L: *P* <0.01, *n* = 9; paired *t*-test, ACh *vs* Bef). **G** Summary of ACh effects on the average duration of spontaneous reverberation (1 μmol/L: *P* = 0.96, *n* = 11; 5 μmol/L: *P* <0.05, *n* = 8; 20 μmol/L: *P* <0.01, *n* = 8; paired *t*-test, ACh *vs* Bef).

In addition to evoked reverberation, spontaneous activity was also recorded in the networks of cultured neurons. In many cases, such activity occurred as spontaneous reverberation (persisting >0.5 s), with repeated polysynaptic activation as in the evoked reverberation. This may be initiated by a few very strong synaptic connections with the help of a background current. When studying evoked reverberation, we generally chose small isolated networks with very low levels of spontaneous activity [26]. However, in some of these networks, such as that shown in Fig. 2A, we recorded a marked increase in spontaneous reverberatory activity after the application of 5 µmol/L ACh that simultaneously suppressed evoked reverberation (Fig. 2A). Further analyses of spontaneous activity in such networks revealed that the application of 5 µmol/L ACh caused a significant increase in the frequency of occurrence of spontaneous reverberation (Fig. 2B), but no significant change in the average duration (Fig. 2C). This effect cannot be attributed only to the competition of synaptic resources between evoked and spontaneous reverberation, as similar enhancement was also induced by 5 µmol/L and 20 µmol/L (but not 1 µmol/L) ACh in larger networks with more baseline spontaneous activity and no evoked reverberation (Fig. 2D–G). Furthermore, a higher dose of ACh (20 µmol/L) not only resulted in higher occurrence, but also increased duration of spontaneous reverberation. Overall, higher doses of ACh appear to make the networks more excitable.

### ACh Inhibits Excitatory, but Not Inhibitory Synaptic Transmission

**Fig. 3:**
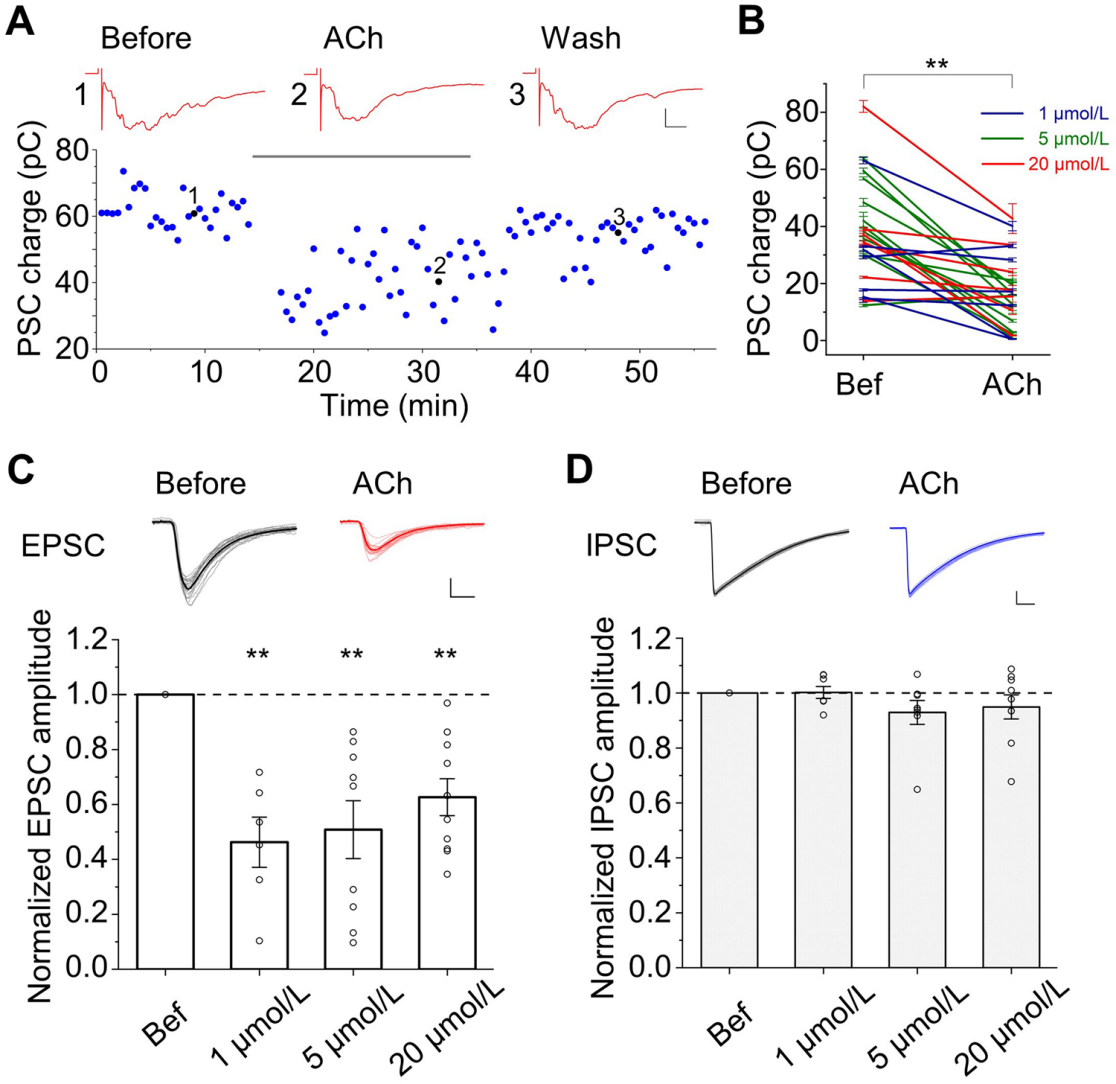
ACh suppresses polysynaptic currents of evoked reverberation as well as monosynaptic EPSCs but not IPSCs. **A** Total charge of the first polysynaptic current (PSC) group decreases after applying 1 μmol/L ACh onto a network; the black horizontal bar indicates the period of ACh application. Inset showing three example traces (1 from “Before”, 2 from “ACh”, and 3 from “Wash”), scale bars, 0.5 nA, 10 ms. **B** Summary of significant ACh-induced decrease in charge of the first PSC group (from 36.98 ± 3.52 pC to 16.30 ± 2.45 pC, *P* <0.001, *n* = 25, paired *t*-test). **C** ACh at various doses significantly suppresses EPSC amplitude (normalized to the value before ACh application. 1 μmol/L: 0.46 ± 0.09, *P* <0.01, *n* = 6; 5 μmol/L: 0.51 ± 0.11, *P* <0.01, *n* = 9; 20 μmol/L: 0.63 ± 0.07, *P* <0.01, *n* = 10; paired *t*-test, ACh *vs* before). Insets are 20 example traces of EPSCs (light gray) and the average trace (black) from before and in the presence of 20 μmol/L ACh (red), respectively. Scale bars, 0.1 nA, 5 ms. **D** As in (**C**) except for IPSC amplitude (1 μmol/L: 1.00 ± 0.02, *P* = 0.92, *n* = 7; 5 μmol/L: 0.93 ± 0.04, *P* = 0.15, *n* = 8; 20 μmol/L: 0.95 ± 0.04, *P* = 0.28, *n* = 9; paired *t*-test, ACh *vs* before). Scale bars, 0.2 nA, 10 ms.

What cellular mechanisms might underlie the modulation of network reverberation by ACh? By examining the polysynaptic current (PSC) traces of reverberatory activity, we noted that the size of the PSCs was reduced in the presence of ACh (Fig. 3A upper panels and S2). A reverberation episode typically consisted of multiple PSCs with some variation in size and pattern. We analyzed the first PSC, which is usually the most stable PSC of a reverberatory trace, and calculated its total charge as a measure of the initial synaptic activation in a reverberation. Indeed, ACh consistently and significantly suppressed the total charge of the first PSC (Fig. 3A lower panel and B). Such effects are likely due to the inhibition of synaptic transmission by ACh. To test this, we made whole-cell recordings of monosynaptic currents in cultured hippocampal neurons, and found that the amplitude of the EPSC was significantly reduced in the presence of ACh (Fig. 3C). In contrast, the amplitude of the inhibitory postsynaptic current (IPSC) was not significantly affected (Fig. 3D). In the network where inhibitory inputs were blocked by the γ-aminobutyric acid type A (GABA_A_) receptor antagonist bicuculline, ACh still suppressed the occurrence probability of evoked reverberation (Fig. S3). These results suggested that the ACh suppression of reverberation is mainly due to its inhibition of excitatory synaptic transmission.

### ACh Increases Neuronal Excitability

**Fig. 4:**
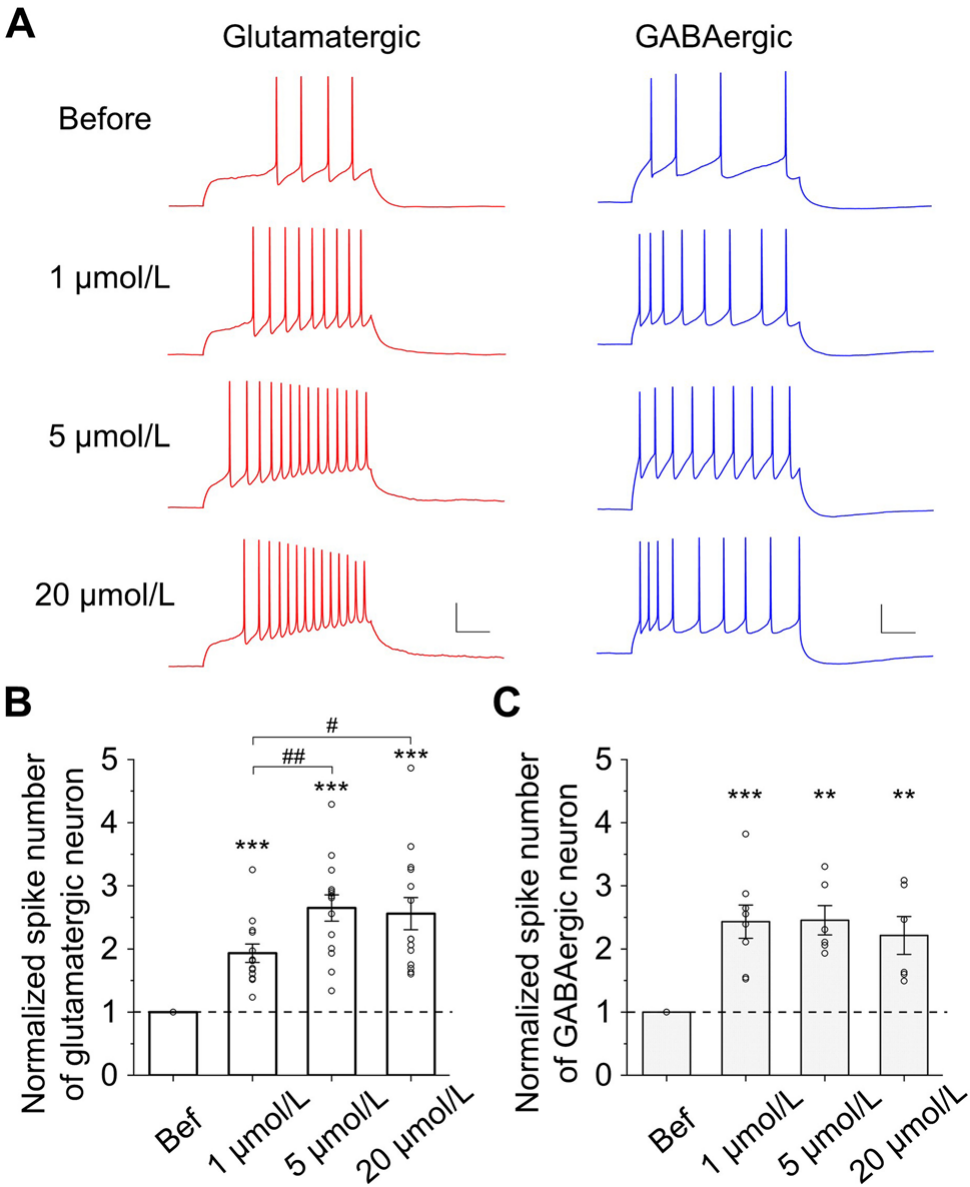
ACh increases neuronal excitability. **A** Example traces of action potentials induced by a depolarizing current injection (500 ms) into a glutamatergic neuron (red) and a GABAergic neuron (blue) under 4 different conditions: before, 1 μmol/L, 5 μmol/L, and 20 μmol/L ACh. Scale bars, 20 mV, 100 ms. **B** ACh increases the number of spikes in glutamatergic neurons (normalized to the value before ACh application. 1 μmol/L: 1.93 ± 0.15, *P* <0.001, *n* = 13; 5 μmol/L: 2.65 ± 0.21, *P* <0.001, *n* = 14; 20 μmol/L: 2.56 ± 0.25, *P* <0.001, *n* = 14; ACh *vs* before, paired *t*-test. 1 μmol/L *vs* 5 μmol/L: *P* <0.01; 1 μmol/L *vs* 20 μmol/L: *P* <0.05; 5 μmol/L *vs* 20 μmol/L: *P* = 0.79; unpaired *t*-test). **C** ACh increases the normalized number of spikes in GABAergic neurons (1 μmol/L: 2.43 ± 0.26, *P* <0.001, *n* = 8; 5 μmol/L: 2.45 ± 0.23, *P* <0.01, *n* = 6; 20 μmol/L: 2.21 ± 0.30, *P* <0.01, *n* = 6; ACh *vs* before, paired *t*-test. 1 μmol/L *vs* 5 μmol/L: *P* = 0.95; 1 μmol/L *vs* 20 μmol/L: *P* = 0.60; 5 μmol/L *vs* 20 μmol/L: *P* = 0.54; unpaired *t*-test).

The excitation of a network depends on both the synaptic connections among neurons and the excitability of each individual neuron. Because ACh inhibited EPSCs (Fig. 3C) and did not affect IPSCs (Fig. 3D), the enhancement of spontaneous network activity by ACh is likely due to increased neuronal excitability. Indeed, in the presence of 1, 5, and 20 µmol/L ACh, the number of spikes increased significantly in both glutamatergic and GABAergic neurons upon step depolarization (Fig. 4A). Interestingly, 5 and 20 µmol/L ACh induced significantly more enhancement in the excitability of glutamatergic neurons (2.65 ± 0.21 for 5 µmol/L and 2.56 ± 0.25 for 20 µmol/L) than 1 µmol/L ACh (1.93 ± 0.15; Fig. 4B). In contrast, in GABAergic neurons, all three doses of ACh caused a similar enhancement in excitability (Fig. 4C). In keeping with more spike firing, ACh caused a slow depolarization of the membrane potential in most of the neurons (Fig. S4A, B), but did not change the input resistance (Fig. S4C, D). These results could, at least in part, explain the enhancement of spontaneous network activity, as well as the increase in evoked reverberation duration in the presence of high doses of ACh.

### Modulation of Reverberation Is Mediated by Muscarinic ACh Receptors

**Fig. 5:**
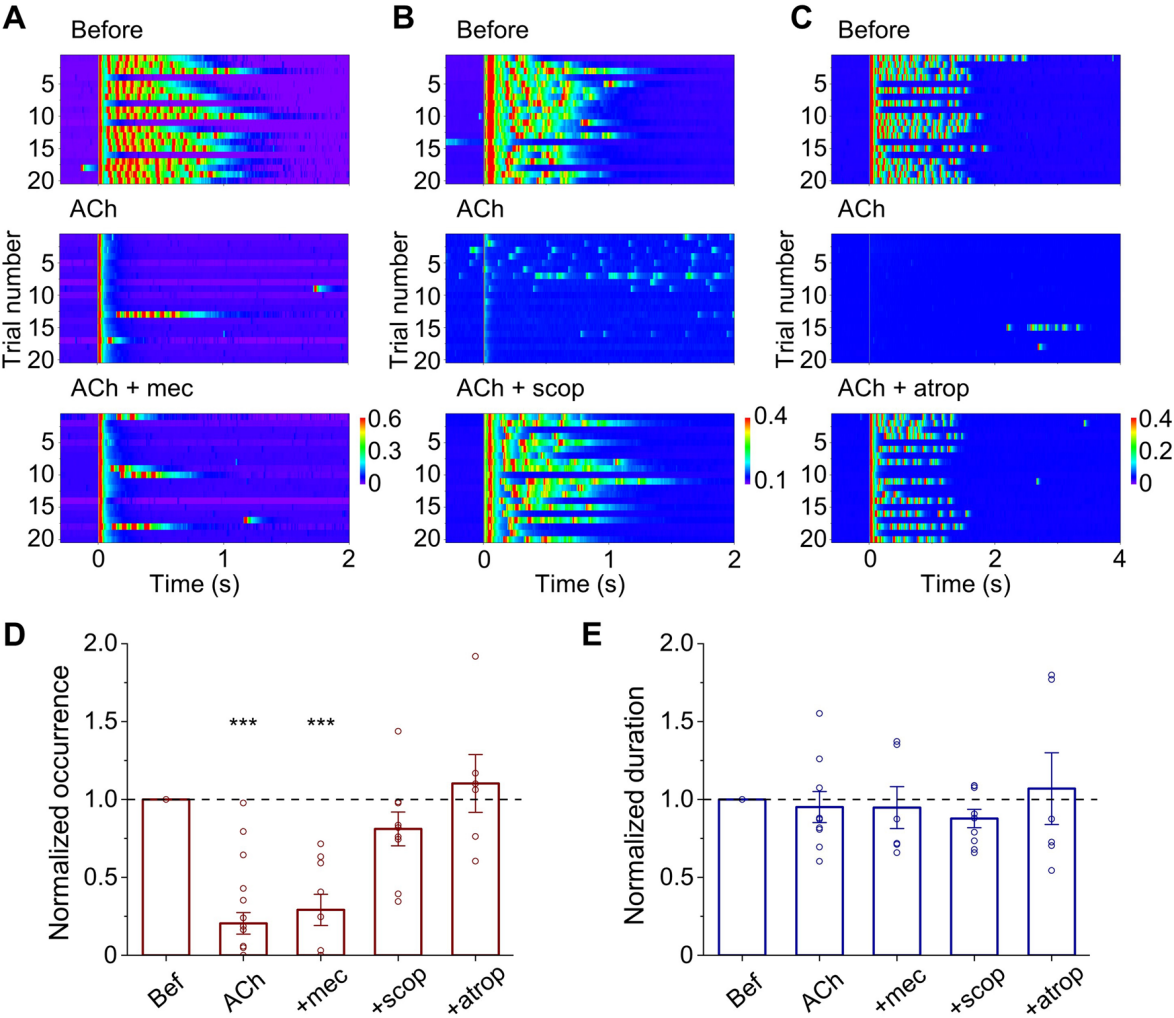
ACh modulates evoked reverberation *via* mAChR signaling. **A–C** Upper: Reverberatory activity elicited in example networks before ACh application. Middle: The occurrence of evoked reverberation is suppressed in the presence of 5 μmol/L ACh. Lower: The ACh-induced decrease in evoked reverberation is rescued by the mAChR antagonists scopolamine (scop, 10 μmol/L) (**B**) and atropine (atrop, 10 μmol/L) (**C**), but not by the nAChR antagonist mecamylamine (mec, 10 μmol/L) (**A**). Pseudo-color represents the current amplitude (in nA). The white vertical bar indicates the period when the ACh-containing solution was being perfused. **D**, **E** Summary of effects of AChR antagonists (10 μmol/L each) on the probability of occurrence (**D**) and duration (**E**) of evoked reverberation in the presence of ACh (5 μmol/L). Normalized occurrence, ACh: 0.20 ± 0.07, *P* <0.001, *n* = 19; ACh + mec: 0.29 ± 0.10, *P* <0.001, *n* = 9; ACh + scop: 0.81 ± 0.11, *P* = 0.31, *n* = 9; ACh + atrop: 1.10 ± 0.19, *P* = 0.69, *n* = 6; unpaired *t*-test, drug *vs* before. Normalized duration, ACh: 0.95 ± 0.10, *P* = 0.62, *n* = 9; ACh + mec: 0.95 ± 0.13, *P* = 0.10, *n* = 6; ACh + scop: 0.88 ± 0.06, *P* = 0.12, *n* = 9; ACh + atrop: 1.07 ± 0.23, *P* = 0.72, *n* = 6; unpaired *t*-test, drug *vs* before.

ACh can modulate neuronal activity through two types of receptors: the ionotropic nicotinic receptor (nAChR) and the G-protein-coupled muscarinic receptor (mAChR). To determine which type of receptor might mediate the modulation of evoked reverberation, we first added the nAChR antagonist mecamylamine together with ACh (5 µmol/L) to neuronal networks, and found that reverberation occurrence still decreased in a manner similar to the addition of ACh alone (Fig. 5A). In contrast, co-application of the mAChR antagonist scopolamine reversed the inhibition of reverberation occurrence by ACh (Fig. 5B). Another classical mAChR antagonist, atropine, also rescued the suppression of reverberation by ACh (Fig. 5C). Summary data show the effects of ACh and different antagonists on reverberation occurrence (Fig. 5D) and duration (Fig. 5E). These three antagonists by themselves did not have any significant effects on reverberation (Fig. S5). These results demonstrated that ACh modulation of evoked reverberation is mediated by mAChRs.

**Fig. 6:**
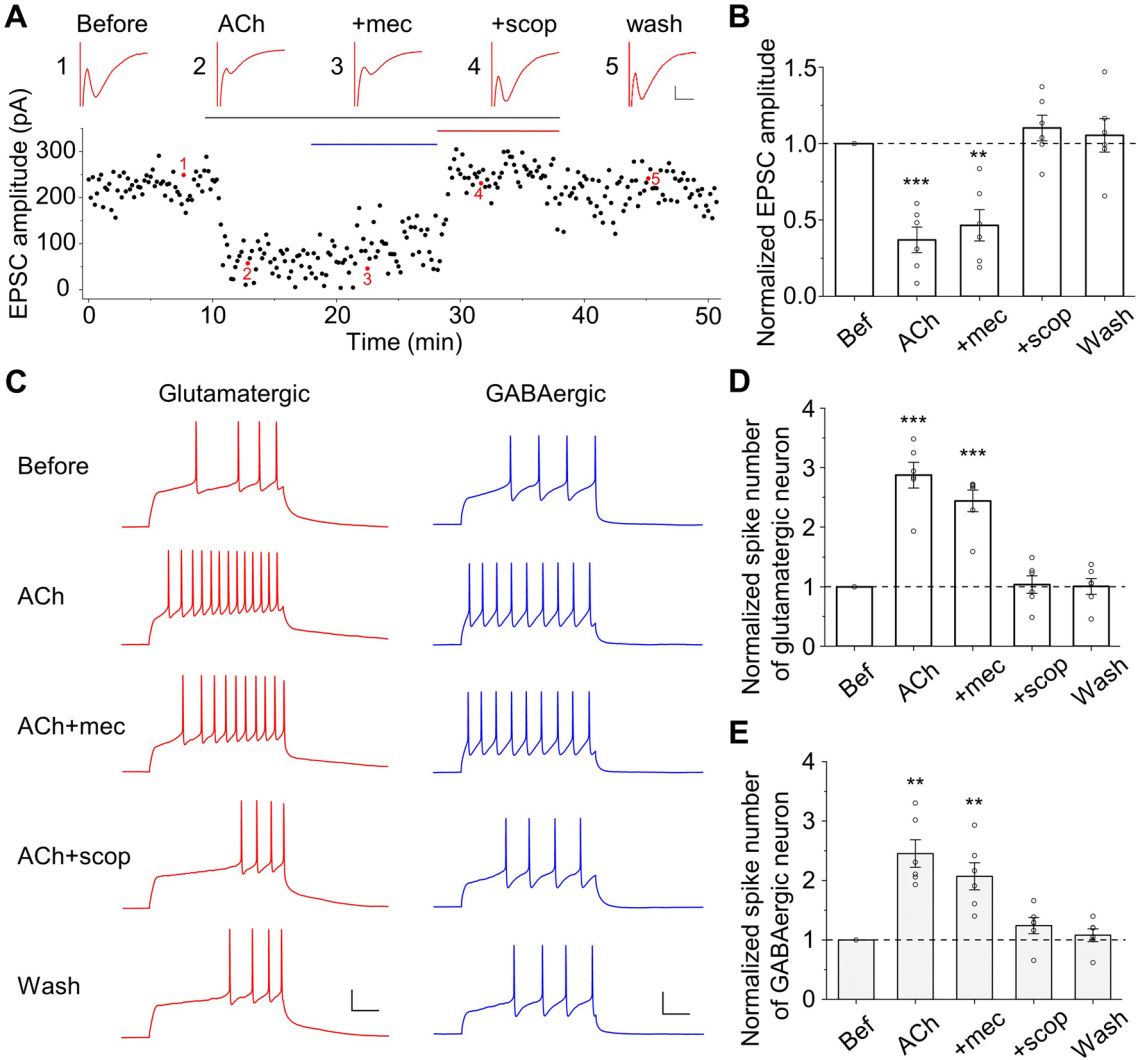
Activation of mAChRs is required for the modulation of synaptic currents and neuronal excitability by ACh. **A** The EPSC amplitude of a neuron changes over time upon application of different drugs (black horizontal bar for 5 μmol/L ACh, blue for 10 μmol/L mecamylamine, and red for 10 μmol/L scopolamine). Example current traces under each condition are shown in the insets. Scale bars, 0.1 nA, 5 ms. **B** Summary of the effects of ACh and AChR antagonists on EPSC amplitude (normalized to the value before ACh application. ACh: 0.37 ± 0.08, *P* <0.001; ACh + mec: 0.47 ± 0.10, *P* <0.01; ACh + scop: 1.10 ± 0.08, *P* = 0.28; Wash: 1.05 ± 0.11, *P* = 0.64; *n* = 6, paired *t*-test, drug *vs* before). **C** Example traces of action potentials induced by a depolarizing current injection (500 ms) into a glutamatergic neuron (red) and a GABAergic neuron (blue). The number of spikes increased by ACh is restored by the mAChR antagonist scopolamine, but not the nAChR antagonist mecamylamine. Scale bars, 20 mV, 100 ms. **D** Summary of effects of ACh and AChR antagonists on the normalized number of spikes in a glutamatergic neuron (ACh: 2.87 ± 0.22, *P* <0.001; ACh + mec: 2.44 ± 0.18, *P* <0.001; ACh + scop: 1.04 ± 0.15, *P* = 0.81; Wash: 1.01 ± 0.13, *P* = 0.96; *n* = 6, paired *t*-test, drug *vs* before). **E** As in **(D)** but for a GABAergic neuron (ACh: 2.45 ± 0.23, *P* <0.01; ACh + mec: 2.07 ± 0.23, *P* <0.01; ACh + scop: 1.24 ± 0.14, *P* = 0.13; Wash: 1.07 ± 0.11, *P* = 0.49; *n* = 6, paired *t*-test, drug *vs* before).

We then determined whether mAChRs are also responsible for the ACh-induced changes in synaptic currents and neuronal excitability. Indeed, both the reduction of EPSC amplitude and the increase of neuronal excitability in the presence of ACh were blocked by scopolamine, but not mecamylamine (Fig. 6). Moreover, the depolarization of the resting membrane potential caused by ACh was also reversed by scopolamine, but not mecamylamine (Fig. S6). Therefore, both the inhibition of excitatory synaptic transmission and the elevation of membrane potential and excitability are mediated by mAChRs.

### *In Silico* Modulation of Network Reverberation

The above experiments naturally pointed to the possibility that the biphasic modulation of network reverberation by ACh was due to its dual action on excitatory synaptic transmission and neuronal excitability. To further test the causal relationship between the altered cellular properties and the modulated network dynamics, we resorted to computational models that have been used to investigate the cross-scale mechanisms of neuromodulation [32, 33]. To this end, we established a biophysical model of networks of neurons that had previously been shown to exhibit reverberatory activity [28]. With this *in silico* system (Fig. S7A), we were able to test whether changes in excitability and EPSC amplitude are sufficient to account for the experimentally recorded modulation of network reverberation by ACh (Fig. S7B–D).

**Fig. 7:**
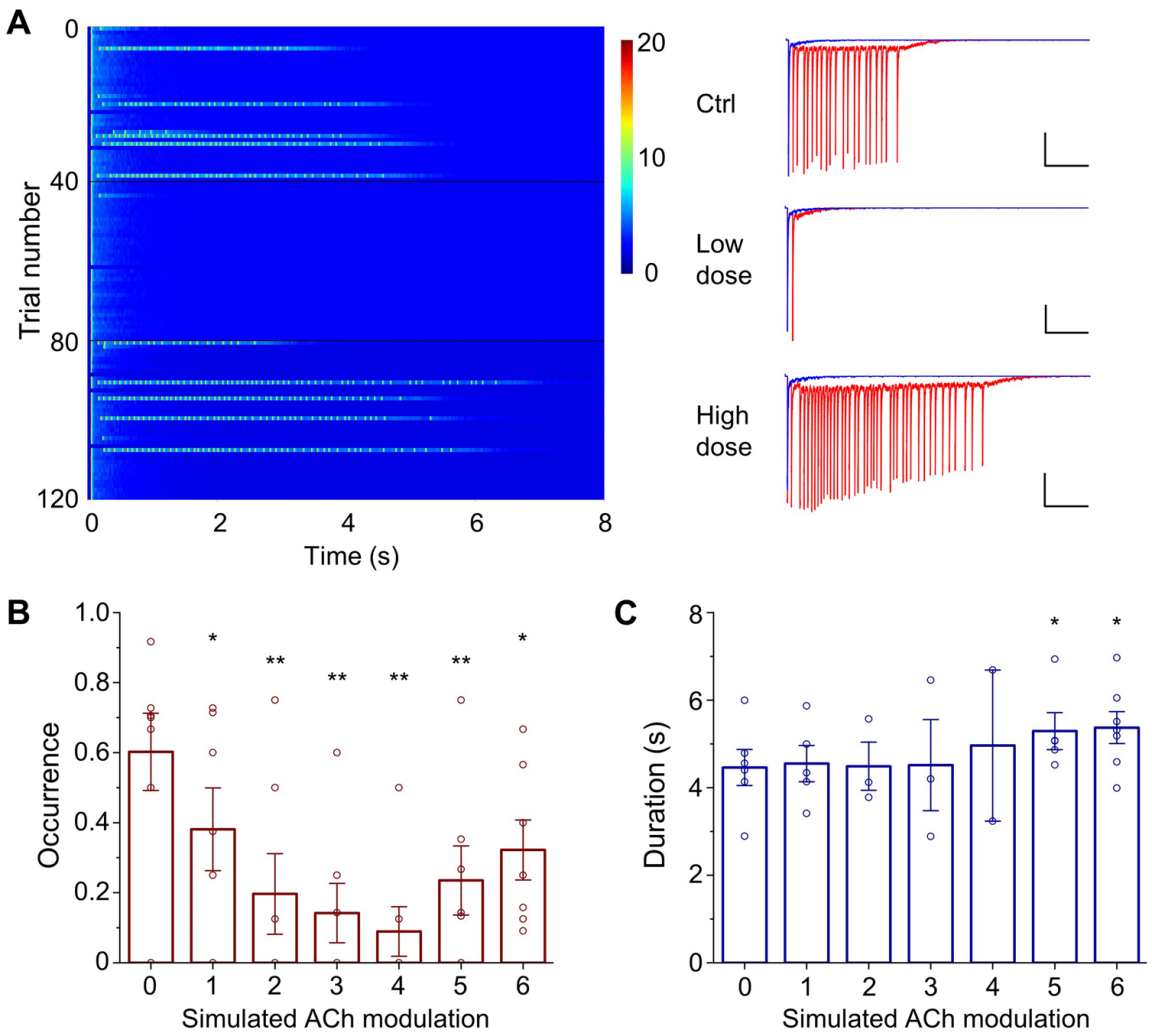
Simulation of biphasic ACh effects on evoked reverberation. **A** Low-dose ACh suppresses evoked reverberation while high-dose ACh enhances reverberation duration. Forty consecutive trials of current traces from three conditions are shown. Trials 1–40: control group (Ctrl); trials 41–80: low-dose ACh; trials 81–120: high-dose ACh. Expanded typical traces in each condition are shown on the right (scale bars, 5 mA, 1 s; blue, short polysynaptic current; red, persistent reverberation). Pseudo-color represents the current amplitude (in mA). **B** Simulated ACh modulation of the occurrence probability of evoked reverberation. Horizontal axis 0–6 represents simulated ACh doses from low to high. Dose-dependent U-shaped modulation was found in the probability of reverberation occurrence (from 0 to 6: 0.60 ± 0.11; 0.38 ± 0.12, *P* <0.05; 0.20 ± 0.12, *P* <0.01; 0.14 ± 0.08, *P* <0.01; 0.09 ± 0.07, *P* <0.01; 0.24 ± 0.10, *P* <0.01; 0.32 ± 0.09, *P* <0.05; *n* = 7, paired *t*-test, “1–6” *vs* “0”). **C** Simulated ACh modulation of the duration of evoked reverberation. Reverberation duration shows an upward tendency (from 0 to 6: 4.46 ± 0.41, *n* = 6; 4.55 ± 0.42, *P* = 0.44, *n* = 5; 4.49 ± 0.55, *P* = 0.24, *n* = 3; 4.51 ± 0.10, *P* = 0.48, *n* = 3; 4.96 ± 0.17, *P* = 0.84, *n* = 2; 5.29 ± 0.42, *P* <0.05, *n* = 5; 5.37 ± 0.36, *P* <0.05, *n* = 7; paired *t*-test, “1–6” *vs* “0”).

For the simulation, we prepared random networks with 20% connectivity sparsity and followed the parameter settings to generate reverberation (Table S1). In order to mimic the experiment, we set the excitability and excitatory synaptic transmission in accordance with different doses of ACh, and recorded the network activity (Fig. 7A). Compared with the control group, the network with alterations in EPSC amplitude and excitability corresponding to a low dose of ACh exhibited a lower occurrence rate of reverberation. In contrast, the cellular parameter sets corresponding to a high dose of ACh showed a higher occurrence probability and longer duration (Fig. 7A). Furthermore, a set of simulations of different ACh doses yielded biphasic modulation of reverberation occurrence, with high doses causing longer reverberation durations (Fig. 7B, C), similar to the experimental results (Fig. 1C, D).

## Discussion

Short-term memory that persists for seconds is believed to be held “online” by persistent neuronal activity [34, 35], perhaps in the form of network reverberation in a group of recurrently-connected neurons, i.e., the “cell assembly” postulated by Donald Hebb [27]. Indications of the existence of a Hebbian cell assembly and properties of its reverberatory activity, such as stimulation-specificity, persistence, synchrony and rhythmicity, and even time sequence, have been reported in different working-memory related *in vivo* experiments [35–39]. However, the enormous complexity of native circuits *in vivo* makes it a very difficult system for the study of how modulation of cellular activity results in relevant changes of network dynamics. In our previous studies, we reported that small networks of cultured neurons that share basic electrophysiological mechanisms with those *in vivo* exhibit persistent network activity with characteristics of Hebbian reverberation [26]. Although care must be taken when interpreting results from such a simplified system, it does provide an accessible system to investigate basic biophysical mechanisms and to link cellular properties with network dynamics. To this end, the current study reveals an interesting dose-dependent feature of how ACh modulates network activity: ACh suppresses the occurrence of evoked network reverberation at low-to-moderate doses, but prolongs reverberation duration and enhances spontaneous network activity at high doses. Such dose-dependent biphasic modulation may also occur *in vivo* with functional consequences. For example, it is possible that attention-related cholinergic activity results in a high ACh concentration in the vicinity of neurons and synapses belonging to specific circuits, thus enhancing their behaviorally relevant reverberatory activity. It is also possible that low concentrations of ACh are due to diffusion to surrounding neurons or synapses belonging to different circuits, thereby suppressing their reverberatory activity and promoting the selectivity of attention or working memory.

It should be noted that, because of spatial constraints, a cultured neuron in a small network tends to form strong, and sometimes suprathreshold, connections with other cells. Consequently, stimulating a single neuron in the network can elicit network reverberation [40]. Interestingly, under certain circumstances, single-cell stimulation indeed elicits ensemble activity in the visual cortex *in vivo* and can even influence animal behavior [39, 41]. In general, however, stimulating a single cultured neuron in a small network may be more analogous to the synchronized activation of multiple neurons in more complex brain circuits. The occurrence of stimuli-specific reverberation might be sensitive to the first evoked synaptic current. On the other hand, it is intuitive that spontaneous reverberation can be readily raised by enhanced network excitability. Indeed, ACh is known to enhance the excitability of hippocampal neurons [24], as confirmed in our experiments (Fig. 4). A closer examination reveals that ACh caused a slow depolarizing current and reduced spike threshold (Figs. S4, S6), probably due to inhibition of the KCNQ family of K^+^ channels [25] that has been shown to cause spontaneous action potential firing [42] and epileptiform bursts [43].

It has been reported that cholinergic suppression of excitatory synaptic transmission in the cortex and hippocampus may affect network activity [44, 45], as also reported in cultured hippocampal neurons (Fig. 3C). This together with the enhanced excitability provides hints for understanding the biphasic effects of ACh on network reverberation. A more quantitative understanding was provided by our *in silico* simulation using a network model based on previous studies [28, 30], which, by setting the EPSC and excitability to follow the same tendency as the biological neurons in ACh experiments, well recapitulated the modulation of reverberatory properties as recorded in cultured neurons. Thus, it is likely that the main driving factors behind the change of reverberatory properties (Fig. 1) are the EPSC and excitability. Furthermore, all the details in the *in silico* model can be measured or altered at each time step, which helps us to isolate how and why the factors at different ACh concentrations affect the occurrence and duration of reverberation. At a low ACh concentration, the EPSC decreased significantly, thus an external stimulus to a single cell could not reliably propagate to the downstream neurons. This led to a significant drop in the occurrence probability of reverberation. The network can still generate reverberation even in the low EPSC situation because once some of the direct downstream neurons of the first stimulated neuron have been excited, some other neurons in the network likely receive more than one (first plus direct downstream) synaptic input in a short time, and thus can be fired reliably. With such chained input amplification, the rest of the neurons can be ignited normally despite the decrease of the EPSC, although the occurrence of reverberation is lower. At higher ACh concentrations, the EPSC does not decrease further but the cellular excitability increases more. Therefore, at low ACh conditions, the decrease of EPSC dominates, and under higher ACh conditions the increased excitability compensates for the decrease of the EPSC, resulting in a more frequent occurrence of reverberation. Note that the EPSC amplitude is tuned by the synaptic vesicle release probability (Eq. 3). When the EPSC is low, the synapse releases fewer vesicles per spike. Because the synapse has a limited vesicle pool size, a lower release probability means more sustainable transmission when the synapse is repeatedly activated. This, together with higher neuronal excitability, could support the network to have a longer reverberatory duration as found in simulation and experiments (Figs. 1B, D and 7A, C).

Muscarinic and nicotinic ACh receptors are widely expressed in the hippocampus, and upon activation by ACh can modulate cellular excitability and synaptic transmission [46]. In our culture system, however, most of the modulatory effects of ACh on network reverberation could be ascribed to muscarinic receptors, probably because of the abundant expression of different subtypes of muscarinic receptors in the hippocampus [47]. It is likely that different effects may involve different muscarinic receptor subtypes [48]. For example, it is known that G_i/o_-linked M2/M4 receptors cause presynaptic inhibition of voltage-gated Ca^2+^ channels, whereas G_q/11_-linked M1 type receptors facilitate postsynaptic excitation by inhibiting K^+^ currents [49]. The actions of these subtypes may help to explain the effects of ACh in reducing excitatory synaptic transmission and elevating neuronal excitability, respectively [23, 50]. In addition, the role of nicotinic receptors appeared to be insignificant in our experiments, although it has been reported that activation of nicotinic receptors can regulate hippocampal excitability and plasticity [51], and facilitate oscillation at the theta frequency in hippocampal networks [52]. Such molecular diversity combined with cellular specificity, e.g., differential projection of cholinergic projections from specific medial septal or basal forebrain neurons onto different hippocampal glutamatergic or GABAergic neurons or synapses, would eventually result in delicate circuit complexity, which in turn underlies efficient and precise brain functions.

In this study, we used cultured hippocampal networks as a model system in which to investigate the Hebbian reverberating cell assembly. Neural circuits grown *in vitro* form specific and stereotyped connectivity patterns through activity-dependent self-organization [53], and neural plasticities such as spike-timing-dependent plasticity and short-term plasticity may create or reorganize these neuronal assemblies [54]. The cholinergic system affects synaptic plasticity in hippocampal neurons [55–57] and subsequently synaptic remodeling and assembly formation during network development. In our experiment, the network activity altered by ACh recovered after washout (e.g., Fig. 2A) without any long-lasting effects, probably because we chose mature networks that already had pre-programmed cell assemblies generating steady reverberation. ACh-mediated neural plasticity might not occur or might not have strong effects on the dynamics of such networks. Neuromodulation and synaptic plasticity both contribute to functional cell assembly at different time scales, with ACh causing reversible changes in the dynamic state of mature or stable networks, whereas synaptic plasticity (whether influenced by ACh modulation or not) causes long-lasting changes in neuronal connections and assembly formation. Such mechanisms may participate in flexible response and learning when animals face different behavioral challenges [58].

The cell assembly in a small mature network usually has a conserved spatial-temporal pattern of evoked reverberation, often with rather uniform occurrence probability and duration [26]. In large networks, multiple assemblies or patterns of reverberatory activity may co-exist, each capable of encoding specific information. By differentially reducing synaptic strength while enhancing neuronal excitability, ACh may dynamically reconstruct the network and selectively alter neural information encoding and retrieval. This could fit in a systems-level scenario where a tonic low level of ACh is involved in setting the global network state, and a phasic high level of ACh is related to learning and attention [59]. It will be interesting to further investigate changes in assembly activity caused by different modes of cholinergic modulation in the context of memory encoding and extraction. Along this line, the very basic properties of network activity and the modulatory effects of ACh found in a simple culture system could help explore such possibilities and understand the behavior of native circuits.

## Supplementary Information

Below is the link to the electronic supplementary material.

Supplementary file1 (PDF 1,163 kb)
