# Supplementary material for: Biphasic Cholinergic Modulation of Reverberatory Activity in Neuronal Networks
Source: Neurosci Bull. 2023 Jan 21;39(5):731–44. doi: 10.1007/s12264-022-01012-7 (PMC10170002; doi:10.1007/s12264-022-01012-7)
Supplement: Supplementary file 1 — Supplementary file1 (PDF 1,163 kb) [file 12264_2022_1012_MOESM1_ESM.pdf]

Supplementary Materials

Supplementary Figures

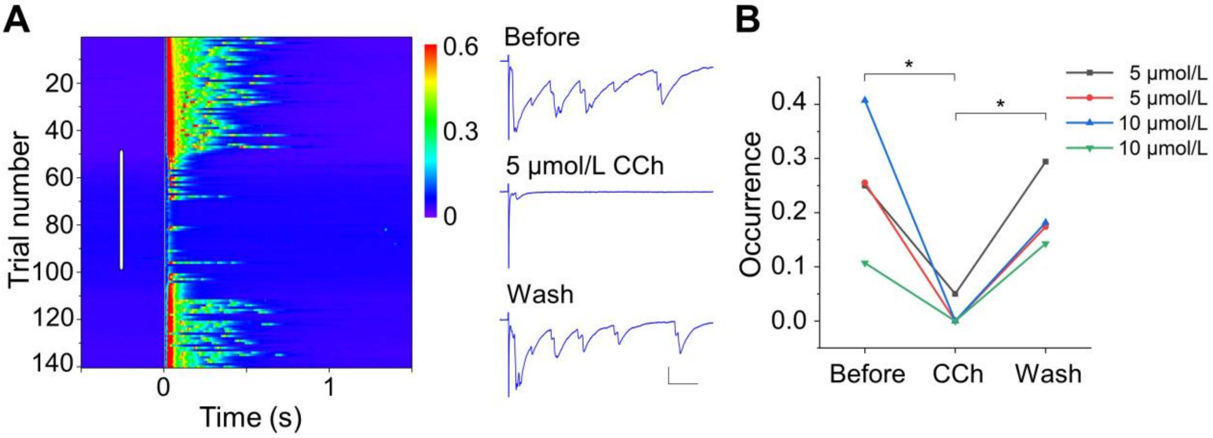

Fig. S1 Carbachol (CCh; 5 μmol/L) suppresses the probability of occurrence of evoked reverberation. **A** Current traces of consecutive trials; the white vertical bar indicates the period from CCh application to wash-out. Typical expanded traces at each stage are shown on the right panel (scale bars, 0.2 nA, 0.1 s). **B** Summary of the effects of CCh (5–10 μmol/L) on the probability of occurrence of evoked reverberation. A significant reduction occurs after the application of CCh (Before, 0.25 ± 0.06; CCh, 0.013 ± 0.013; Wash, 0.20 ± 0.03; n = 4, P <0.05, paired t-test).

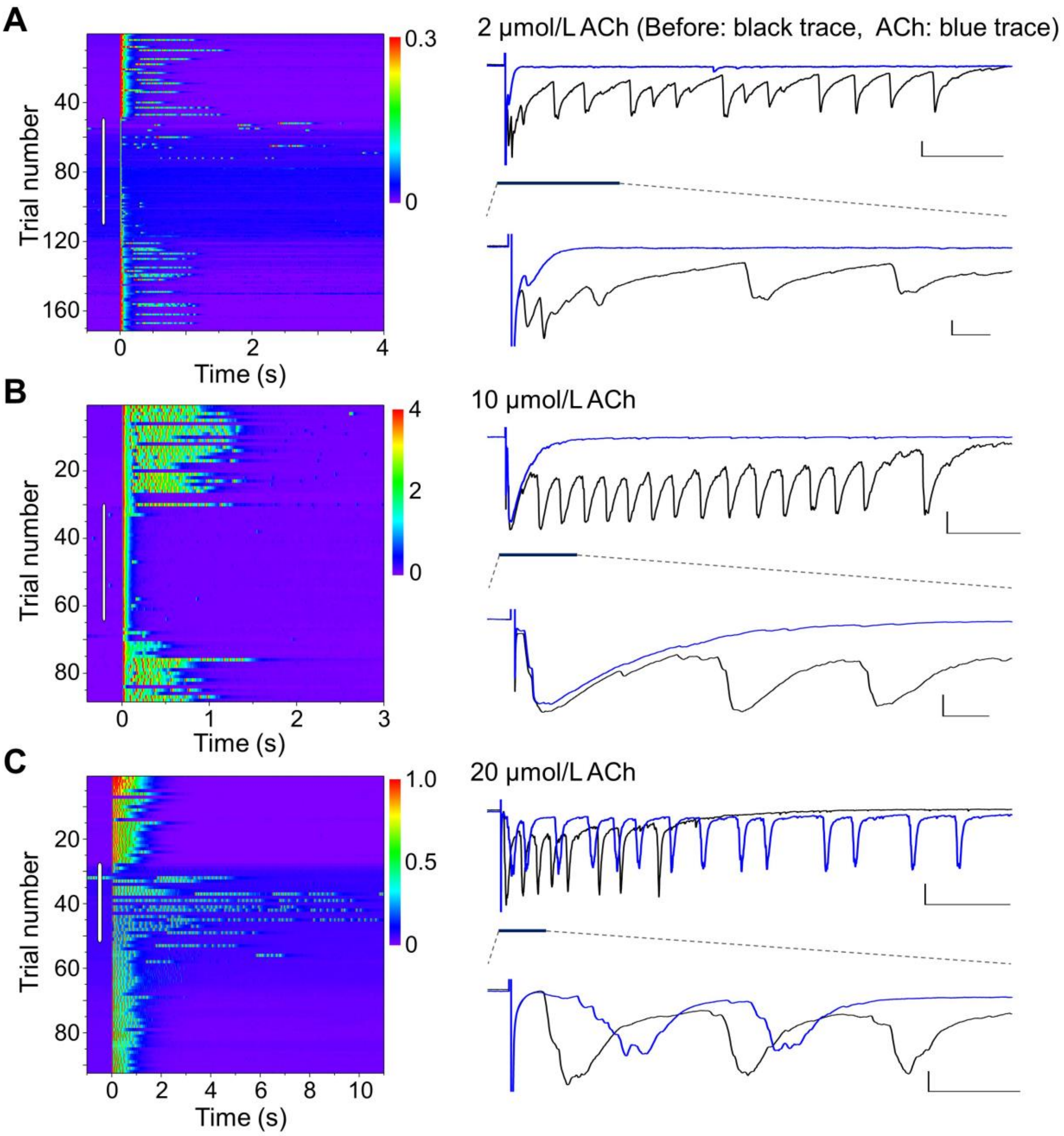

Fig. S2 ACh suppresses polysynaptic currents of evoked reverberation. **A** Example network showing the effects of 2 μmol/L ACh. Left: 2 μmol/L ACh (indicated by the white vertical bar) inhibits reverberation. Right: example traces before (black) and after ACh (blue). Scale bars: upper panel, 0.1 nA, 0.2 s; lower panel, 0.1 nA, 20 ms. **B** Example network showing the effects of 10 μmol/L ACh. Scale bars: upper panel, 1 nA, 0.2 s; lower panel, 1 nA, 20 ms. **C** Example network showing the effects of 20 μmol/L ACh. Scale bars: upper panel, 0.5 nA, 0.5 s; lower panel, 0.5 nA, 50 ms.

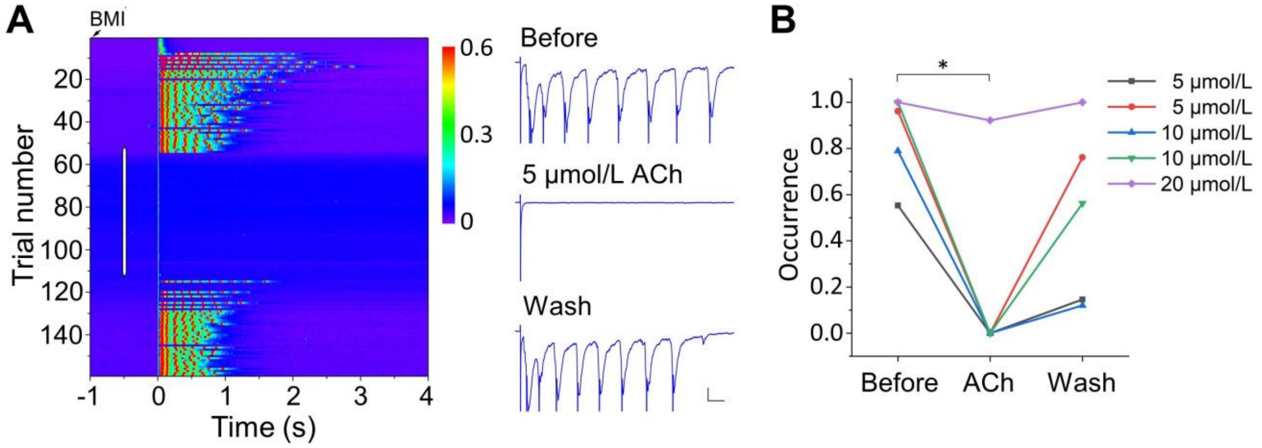

Fig. S3 ACh suppresses the probability of occurrence of evoked reverberation in the presence of a $GABA_A$ receptor antagonist. **A** ACh (5 μmol/L) suppresses evoked reverberation in the presence of 10 μmol/L bicuculline methiodide (BMI). Left: current traces of consecutive trials; the white vertical bar indicates the period from ACh application to wash-out. Right: typical expanded traces of each stage (scale bars: 0.2 nA, 0.1 s). **B** Summary of the effects of ACh (5–20 μmol/L) in the presence of BMI on the probability of occurrence of evoked reverberation. A significant reduction occurs after the application of ACh (Before: 0.86 ± 0.09, ACh: 0.18 ± 0.18, Wash: 0.52 ± 0.17, $n = 5$, $P < 0.05$, paired *t*-test).

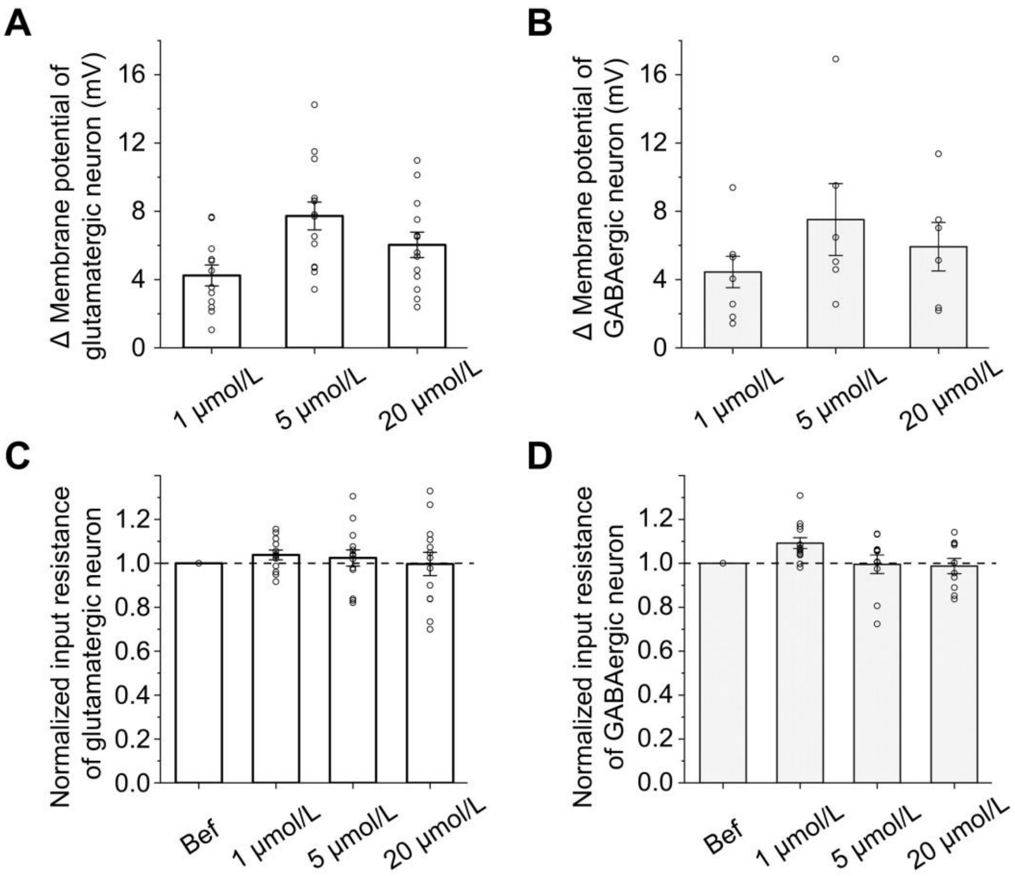

Fig. S4 ACh depolarizes the neuronal membrane potential but does not affect input resistance. **A** Change of membrane potential ($V_{ACh} - V_{bef}$) of glutamatergic neurons after application of ACh. Neuronal membrane potential showed depolarization at all test doses (1 µmol/L: 4.24 ± 0.61, 5 µmol/L: 7.73 ± 0.82, 20 µmol/L: 6.74 ± 0.74). **B** Change of the membrane potential of GABAergic neurons after application of ACh (1 µmol/L: 4.46 ± 0.92, 5 µmol/L: 7.51 ± 2.10, 20 µmol/L: 5.93 ± 1.42). **C** The normalized input resistance of glutamatergic neurons does not change after ACh. 1 µmol/L: 1.04 ± 0.02, 5 µmol/L: 1.02 ± 0.04, 20 µmol/L: 1.00 ± 0.05. **D** The normalized input resistance of GABAergic neurons does not change much (<10%) after ACh (1 µmol/L: 1.09 ± 0.02, 5 µmol/L: 1.00 ± 0.04, 20 µmol/L: 0.99 ± 0.03).

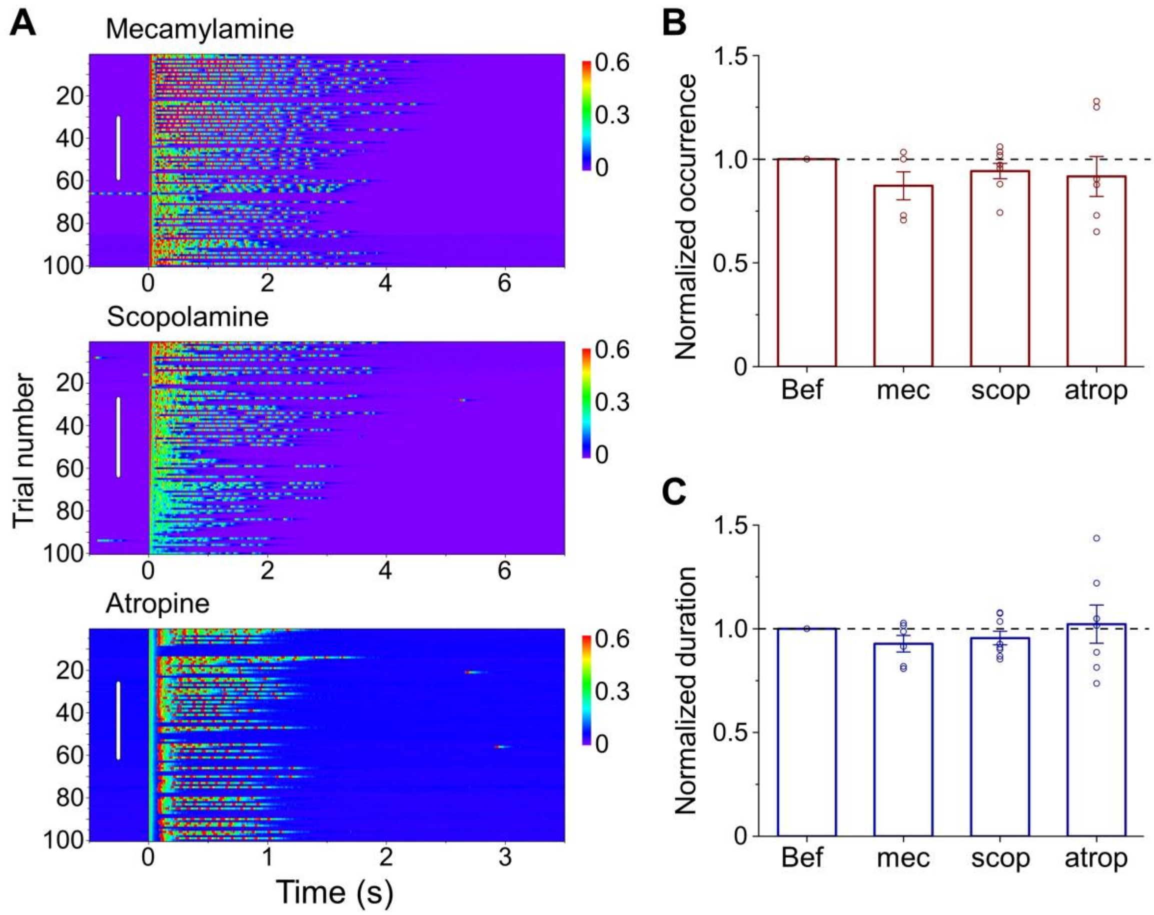

Fig. S5 ACh receptor antagonists by themselves have no effect on evoked reverberation. **A** Application of mecamylamine (mec), scopolamine (scop), or atropine (atrop) (10 μmol/L each) has no effect on evoked reverberation. White vertical bars indicate periods of drug application. Pseudo-color represents the current amplitude (in nA). **B** Summary of the effects of antagonists on the occurrence of evoked reverberation (normalized to values before drug application. mec: 0.87 ± 0.07, n = 6, P = 0.15; scop: 0.94 ± 0.03, n = 8, P = 0.24; atrop: 0.92 ± 0.10, n = 7, P = 0.48; paired t-test, drug *vs* before). **C** Effects of antagonists on the normalized duration of evoked reverberation (mec: 0.92 ± 0.04, n = 6, P = 0.14; scop: 0.96 ± 0.03, n = 6, P = 0.43; atrop: 1.02 ± 0.09, n = 6, P = 0.97; paired t-test, drug *vs* before).

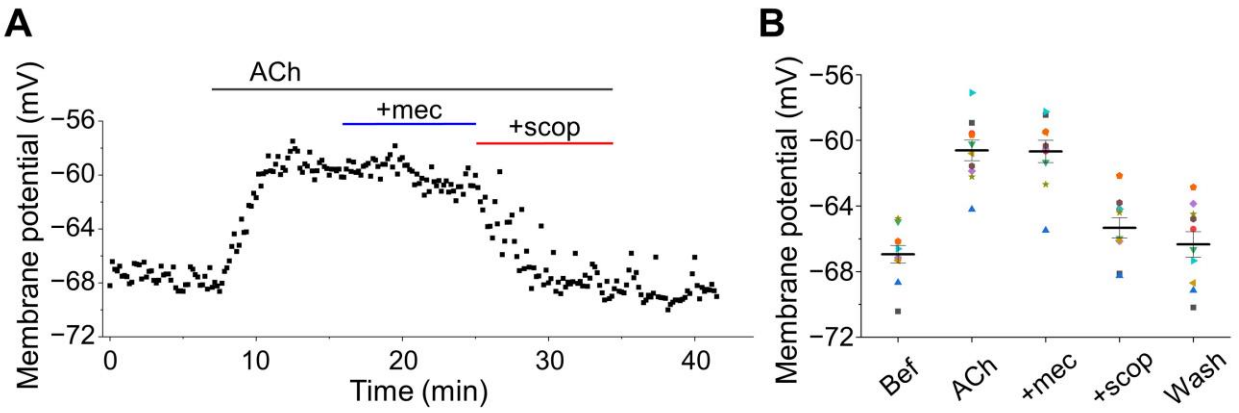

Fig. S6 ACh causes membrane depolarization *via* muscarinic acetylcholine receptor (mAChR) signaling. **A** Example membrane potential recording showing ACh-induced depolarization and restoration by mAChR antagonists. Black, blue, and red lines indicate the application of 5 μmol/L ACh, ACh with 10 μmol/L mecamylamine, and ACh with 10 μmol/L scopolamine, respectively. **B** Summary of membrane potential in 5 μmol/L ACh only, ACh with 10 μmol/L mecamylamine, ACh with 10 μmol/L scopolamine, and washout. Colored data points are the average membrane potential values within corresponding periods recorded from each neuron. Thick black bars indicate the mean membrane potential values of all neurons. Error bars, SEM (Before: 66.95 ± 0.53 mV; ACh: 60.61 ± 0.63 mV, P <0.001; ACh + mec: 60.67 ± 0.68 mV, P <0.001; ACh + scop: 65.33 ± 0.61 mV, P <0.01; Wash: 66.34 ± 0.78 mV, P = 0.32; n = 10, paired t-test, drug *vs* before).

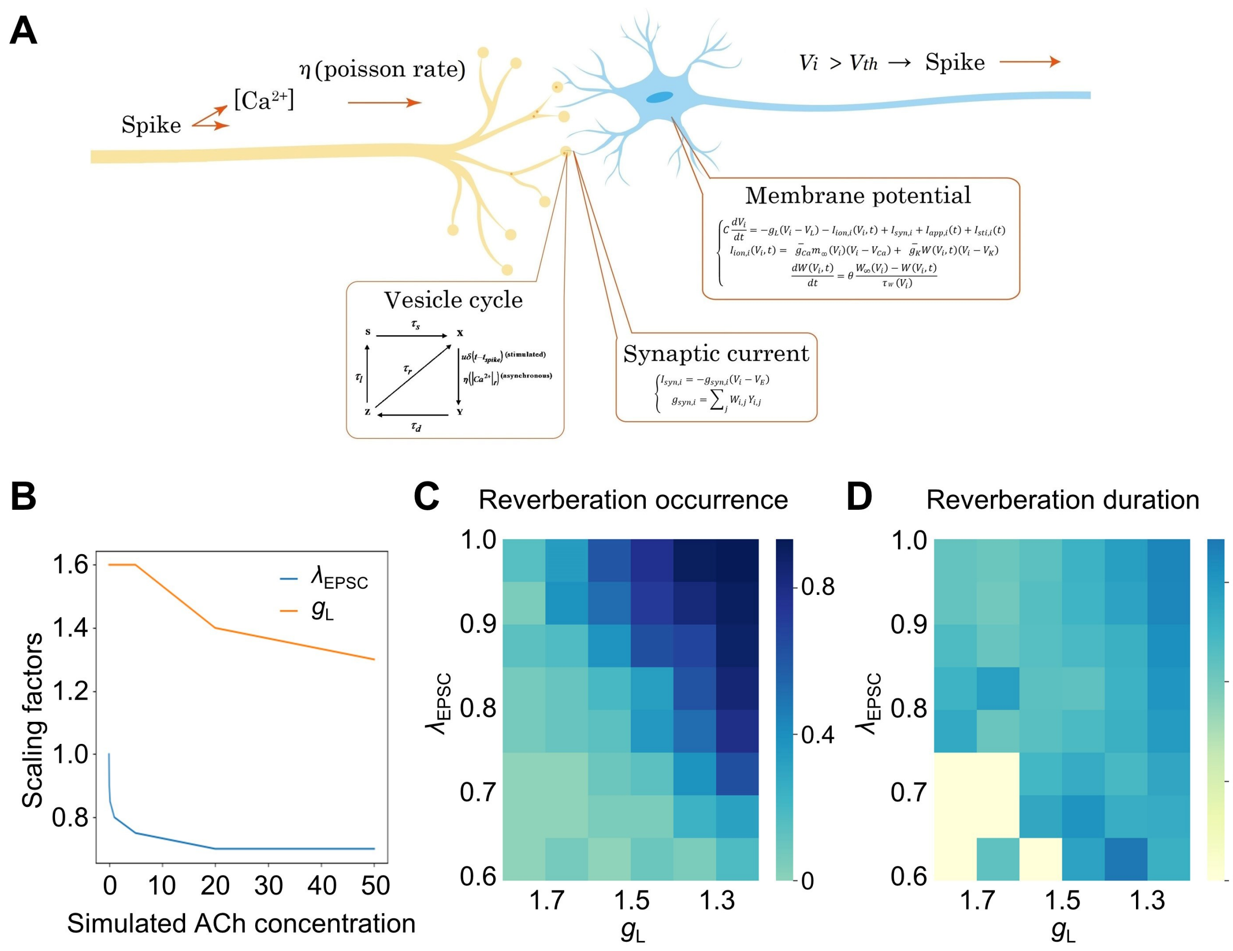

Fig. S7 Illustration of reverberation simulation. **A** Schematic of the neuronal network model. The model simulates the dynamics of the vesicle cycle, synaptic current, and membrane potential, which can modulate network reverberation. **B** Tuning of λ_{EPSC} and g_{L} along with increasing ACh concentration in arbitrary units, corresponding to ACh doses from zero (0) to super high (50). **C**, **D** 2D parameter scan of λ_{EPSC} and g_{L}; colored patches denote the values of the simulated probability of the occurrence of reverberation (**C**) and duration (**D**). In the simulation, EPSC is positively correlated with λ_{EPSC}, and neuronal excitability is negatively correlated with g_{L}. As λ_{EPSC} and g_{L} decline a pathway that fits the experimental phenomena can be found.

Supplementary Tables

Table S1 Parameters used in simulations

Parameter	Value	Description
g_{Ca}	4.4 mS/cm^2	Conductance of Ca^{2+} channel
g_K	8 mS/cm^2	Conductance of K^+ channel
g_L	2 mS/cm^2	Conductance of leak channel
V_{Ca}	120 mV	Equilibrium potential of Ca^{2+} channel
V_K	−84 mV	Equilibrium potential of K^+ channel
V_L	−60 mV	Equilibrium potential of leak channel
V_1	−1.2 mV	Turning point of the voltage-dependent function of activation variable *m*
V_2	18 mV	Span of the voltage-dependent function of activation variable *m*
V_3	2 mV	Turning point of the voltage-dependent function of activation variable *W*
V_4	30 mV	Span of the voltage-dependent function of activation variable *W*
V_E	0 mV	Equilibrium potential of excitatory synapse
θ	0.04	Reference frequency
C	2 μF/cm^2	Membrane capacitance
τ_d	10 ms	Time constant of postsynaptic current decrease
τ_r	61 ms	Time constant of inactive state vesicle transform to recovered state

τ_I	1250 ms	Time constant of inactive state vesicle transform to super-inactive state
τ_s	10 s	Time constant of super inactive state vesicle transform to recovered state
μ	0.25	Quantity of neurotransmitter release per spike
I_{app}	3.5 mA	Background current input in neurons

Table S2 Variables that affect neuronal excitability

Variables	Value	Derivative
g_{Ca}	4.4	−10.5801
g_K	8	2.958213
g_L	2	70.8396
V_{Ca}	120	−0.22268
V_K	−84	−0.53797
V_L	−60	−3.80167
V_1	−1.2	9.449592
V_2	19	−14.7947
V_3	2	−4.83498
V_4	30	7.642972
